# Analysis of the efficacy of adnexal mass diagnosis by senior and junior sonologists based on subjective diagnosis and O-RADS classification

**DOI:** 10.1186/s12905-026-04487-y

**Published:** 2026-04-25

**Authors:** Anyan Zhou, Rong Hu, Xin Liu, Gulibibu Kuyeyibieergan

**Affiliations:** Department of Obstetric Ultrasound, Xinjiang Medical University First Affiliated Hospital, Xinjiang Uygur Autonomous Region Urumqi, 830011 China

**Keywords:** O-RADS, Subjective diagnosis, Ovarian neoplasms, Ultrasonography

## Abstract

**Background:**

In clinical practice, the majority of adnexal masses(AMs) are assessed and diagnosed by junior ultrasonologists, as there are more primary ultrasonologists than specialists. However, diagnostic omissions and misdiagnoses are common due to the inexperience of junior ultrasonologists, and diagnostic accuracy varies significantly among different individuals and research centres. Therefore, a more reliable assessment of the malignancy risk of adnexal lesions is necessary to aid sonologists, particularly those who lack experience, in diagnosing AMs.The purposes of this study were to compare the effectiveness of benign and malignant adnexal mass diagnosis based on subjective assessments and the O-RADS US among sonologists with different experience levels and evaluate consistency; furthermore, to analyse the reasons for missed and misdiagnosed cases and inconsistent O-RADS classification to promote the practical application and promotion of O-RADS ultrasound classification in clinical settings.

**Methods:**

A senior and a junior sonologist subjectively evaluated and O-RADS classified adnexal masses using the blind method, both of them had participated in the systematic learning and training of O-RADS diagnostic guidelines. With postoperative pathological results as the gold standard, the receiver operating characteristic curve was used to test the diagnostic performance. Kappa (k) statistics were used to assess the interobserver agreement.

**Results:**

Of the 530 adnexal lesions, 470 (88.7%) were benign and 60 (11.3%) were malignant. The area under the curve of O-RADS classification and subjective judgment were 0.94 (95% CI: 0.92–0.96) and 0.79 (95%CI: 0.73–0.84) for the junior sonologist (*P* < 0.05) and 0.96 (95% CI: 0.94–0.97) and 0.93 (95% CI: 0.88–0.98) for the senior sonologist (*P* < 0.05). The interobserver agreement was poor in subjective judgment (Kappa value was 0.34, *P* < 0.05). The Kappa value of O-RADS classification was 0.91( *P* < 0.05).

**Conclusions:**

For both the senior and junior sonologists, the diagnostic efficacy and consistency using the O-RADS were better than those of their subjective diagnoses. When the junior sonologist utilized the O-RADS, diagnostic efficacy was significantly improved.

## Background

Based on the latest data from US Cancer Statistics, it is projected that there will be 20,890 newly diagnosed cases of ovarian cancer and 12,730 deaths in the United States in 2025 [1]. Globally, ovarian cancer accounts for approximately 150,000 annual deaths, with a notably high mortality rate [2]. The lack of distinct symptoms and effective screening methods for early-stage ovarian cancer leads to the disease typically not being detected until a late stage, which is the primary cause of the high fatality rate of the disease [3]. Identifying a reliable early detection tool for ovarian tumours is critical. Ultrasound diagnosis of ovarian cancer has high sensitivity and specificity (90%-97%) [4–6], and it is considered the best imaging method for the preoperative diagnosis of benign and malignant adnexal masses (AMs). However, due to the diverse origins of ovarian adnexal tumours and the varying performance of ultrasounds, the diagnosis of these tumours through ultrasound is highly dependent on operator expertise [7]. The evaluation of pelvic mass images by senior ultrasonologists to determine whether AMs are benign or malignant is a common method. However, the accuracy of this method is only 90% [8, 9], indicating the need for further improvement.

In recent years, several ultrasound reporting systems have been used to assess the risk of ovarian tumour malignancy, including the following: the system developed by the International Ovarian Tumor Analysis (IOTA) group in 2000, which proposed terminology and definitions to describe the ultrasound characteristics of adnexal tumours [10]; the 2005 “Logistic Regression Model“ [11]; the 2008 Simple Rules (IOTA SR) [12]; the 2009 “Gynaecologic Imaging Reporting Data System (GI-RADS)“ [13]; the 2014 “ADNEX Model“ [14]; and the 2016 Simple Rules-Risk Model (SR-Risk) [15].In addition, in 2018, the American College of Radiology (ACR) published a white paper describing adnexal lesions [16]. The Ovarian Adnexal Imaging Reporting and Data System (O-RADS) Consensus Guidelines for Ultrasound Risk Stratification and Management were published by the 2020 Ultrasound Working Group [17]. The risk determined by ovarian adnexal ultrasound is categorized into the following 6 classes (O-RADS 0–5) according to the consensus guidelines: O-RADS 0, an incomplete evaluation; O-RADS 1, the physiological category (normal premenopausal ovary); O-RADS 2, the almost certainly benign category (1% risk of malignancy); O-RADS 3, lesions with a low risk of malignancy (1% -10%); O-RADS 4, lesions with an intermediate risk of malignancy (10%-50%); and O-RADS 5, lesions with a high risk of malignancy (≥ 50%). The O-RADS is currently the only standardized lexicon and risk stratification system that includes all risk categories and associated management options.

The complexity of the O-RADS classification system renders it challenging to apply in clinical practice, and inconsistencies may arise when physicians of varying levels of experience utilize the system. In this study, our aim was to investigate the diagnostic efficacy and consistency of O-RADS classification of AMs. The objectives of this study were as follows: (1) to compare the diagnostic efficacy and consistency of sonologists with different experience levels in classifying benign and malignant AMs based on subjective assessment and O-RADS classification; (2) to determine the histopathological types for which inconsistency is present, analyse the possible reasons for the inconsistency, and analyse missed and misdiagnosed cases; (3) to help to promote further improvement of the O-RADS classification system.

## Methods

### Ethical standards compliance

This study received approval from the Ethics Committee of the First Affiliated Hospital of Xinjiang Medical University, which also waived the need for patients’ written informed consent.

### Research sample

We retrospectively analysed data from 530 patients who underwent gynaecological surgery at the First Affiliated Hospital of Xinjiang Medical University between January 2022 and March 2023. The data included complete preoperative ultrasonographic and postoperative pathological results. Complete medical data were obtained for all patients, including their age, gynaecological ultrasonographic images, intraoperative findings, and postoperative pathology. Anonymization was applied consistently to all patient data and images. The patients’ ages ranged from 4 to 88 years, with a median age of 36. For the study, we selected the AM with the highest O-RADS classification if a patient had multiple masses, either unilateral or bilateral. All patients underwent transvaginal or transrectal ultrasound, supplemented by transabdominal ultrasound if the lesion was too extensive. Specialized pathologists at our hospital conducted thorough examinations of the pathological samples and subsequently issued the corresponding results.

The inclusion criteria were as follows: (1) Patients who underwent preoperative ultrasound examination in our hospital and had a time interval of less than 30 days between the examination and gynecological surgery; (2) patients who had not undergone surgery on the adnexal or ovary or radiotherapy; (3) female patients with complete image information that could be classified as O-RADS classes 2–5; and (4) female patients who had a clear pathological diagnosis result after the operation.

The exclusion criteria were as follows: (1) poor quality ultrasound images; (2) the inability to perform the O-RADS classification; (3) patients without complete clinical data; (4) cases classified as category 0 and 1 by O-RADS; and (5) pregnant women with adnexal lesions.

The diagnosing physicians: The junior sonologist had 3 years of experience, while the senior sonologist had 20 years of experience.

### Instrumentation and image analysis

**Fig. 1:**
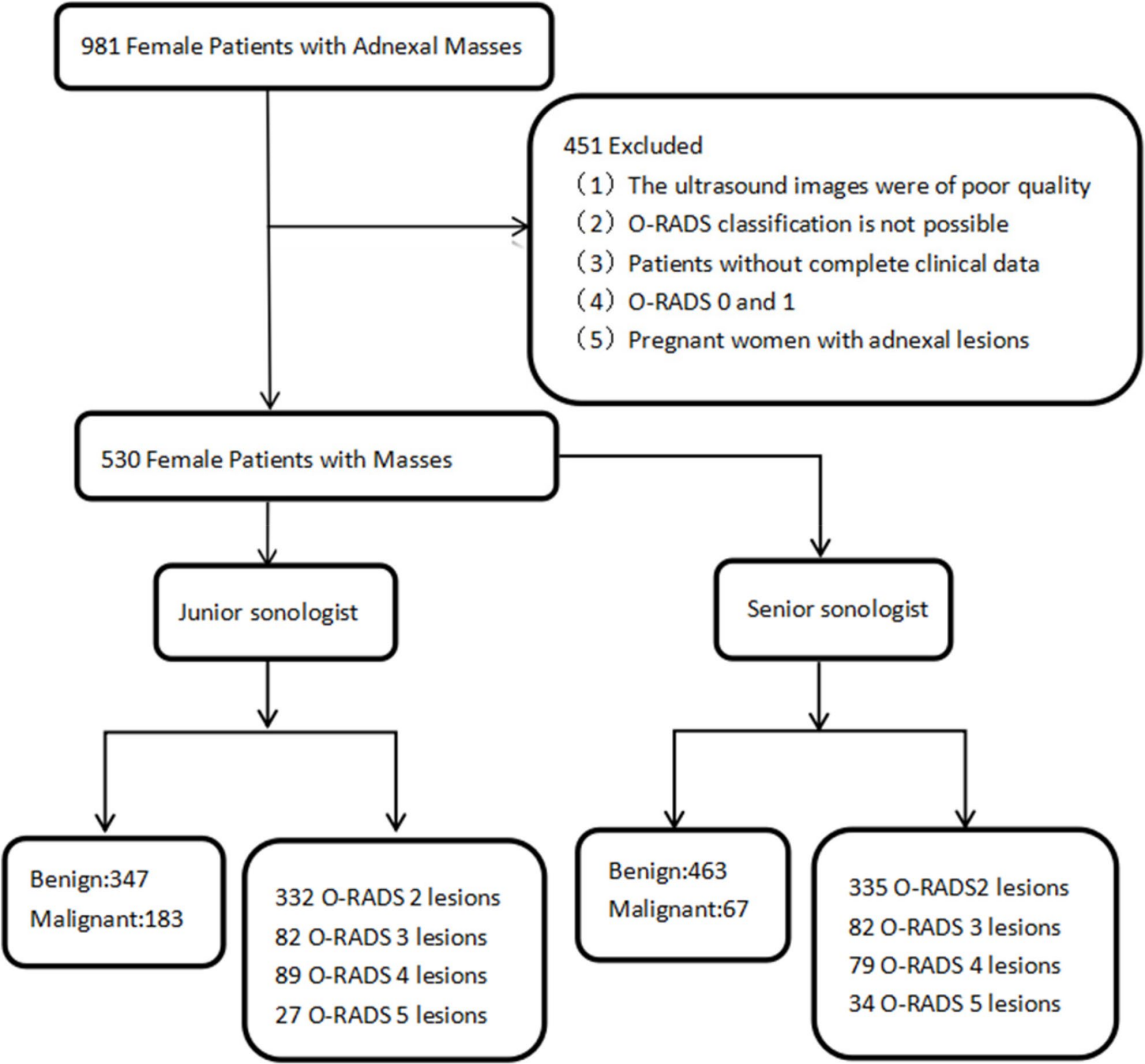
. The flow chart of our study

The GE-E8, SAMSUNG-WS 80, and Mindray DC-8EXP color Doppler ultrasound machines were used, with a transabdominal probe operating at a frequency range of 2.5-5.0 MHz and an intracavitary probe operating at a frequency range of 5–9 MHz. Ultrasound images were obtained from the ultrasound reporting system of the First Affiliated Hospital of Xinjiang Medical University. According to the O-RADS ultrasound dictionary, the primary aim is to observe and document the following: lesion type, maximum diameter, papillary projection or nodule, separation, solid components, external contours, internal echoes of solid or solid-dominant lesions, internal walls and internal echoes of cystic lesions, colour scores, peritoneal fluid, peritoneal thickening and nodules. A senior and junior sonologist with 20 years and 3 years of experience in gynaecological ultrasound diagnosis, respectively, were included in the present study. The sonologists randomly and independently subjectively evaluated AMs without knowledge of the pathological findings and then received theoretical and practical training in O-RADS classification, with lesions classified into O-RADS categories 2, 3, 4, and 5. Lesions classified as O-RADS categories 2 and 3 are considered benign tumours, and those classified as O-RADS categories 4 and 5 are considered malignant tumours. Tumours were classified according to the World Health Organization International Classification of Ovarian Tumors (WHO ICOT), and borderline tumours were classified as malignant tumours. The flow chart of this study is shown in Fig. 1.

## Results

### Patient characteristics and histological type of pathology

A total of 530 patients were enrolled. The average age was 38.8 ± 14.4 years. Postoperative pathology confirmed 470 cases of benign tumours (470/530,89%) and 60 cases of malignant tumours (60/530,11%). A total of 188 tumours were epithelial and mesenchymal tumours, 19 were sex cord-stromal tumours, 93 were germ cell tumours, and 230 were other types of tumours. The most common benign tumour found through pathological analysis was endometriotic cyst of the ovary, whereas the most common malignant tumour was serous carcinoma. The statistics of the pathological results are detailed in Table 1.

**Table 1:** Pathological diagnoses of the 530 adnexal masses

Pathology	*n* (%)	Pathology	*n* (%)
Benign	470 (89)	Malignant	60 (11)
Serous surface papilloma	1	Mucinous carcinoma	2
Serous adenofibroma	7	Mucinous borderline tumour	2
Seromucinous cystadenoma	7	Endometrioid carcinoma	2
Serous cystadenoma	85	Clear cell carcinoma	3
Mucinous cystadenoma	43	Endemetrioid borderline tumour	3
Leiomyoma	1	Serous borderline tumour	12
Leydig cell tumours	1	Serous carcinoma	21
Microcystic stromal tumour	1	Adult granulosa cell tumour	1
Sclerosing stromal tumour	1	Cellular fibroma	2
Fibroma	6	Teratoma with malignant transformation	1
Thecoma	7	Yolk sac tumour	1
Gonadoblastoma	1	Dysgerminoma	2
Mature cystic teratoma	83	Immature teratoma	3
Struma ovarii	2	Other malignant tumors	5
Adnexal inflammatory mass	3		
Follicle cyst	4		
Simple cyst	18		
Ovarian endometriosic cysts	131		
Other benign tumors	68		

### Subjective assessment

When making subjective diagnoses, the junior sonologist diagnosed 347 tumours (347/530,65%) as benign tumours and 183 tumours (183/530,35%) as malignant tumours. On the other hand, senior sonologist diagnosed 463 tumours (463/530,87%) as benign tumours and 67 tumours (67/530,13%) as malignant tumours.

Subjective misdiagnosis by the junior sonologist resulted in a misdiagnosis rate of 28.1%(132/470), with 132 benign lesions being incorrectly judged as malignant. These mainly included mature teratoma (23/83 cases; 27.7%), struma ovarii (1/2 cases; 50%), endometriotic cyst of the ovary (31/131 cases; 23.7%), hydrosalpinx (7/38 cases; 18.4%), torsion of ovarian cyst (3/3 cases; 100%), serous cystadenoma (27/85 cases; 31.8%), and mucinous cystadenoma (15/43 cases; 34.9%).

The junior sonologist missed nine malignant lesions, yielding a 15% (9/60) miss rate. These mainly included one case of teratoma with malignant transformation, four cases of serous borderline tumour, one case of cellular fibroma, one case of endemetrioid borderline tumour, one case of serous carcinoma, and one case of clear cell carcinoma.

Subjective misdiagnosis by the senior sonologist resulted in a misdiagnosis rate of 3%(14/470), with 14 benign lesions being incorrectly judged as malignant. These mainly included one case of mature teratoma, one case of serous cystadenoma, one case of serous adenofibroma, one case of struma ovarii, one case of leiomyoma, one case of microcystic stromal tumour, one case of fibroma, one case of an adnexal inflammatory mass, one case of sclerosing stromal tumour, two cases of mucinous cystadenoma, and three cases of thecoma.

The senior sonologist missed 7 malignant lesions, yielding a 11.7%(7/60) miss rate.These mainly included 4 cases of serous borderline tumour, one case of clear cell carcinoma, one case of teratoma with malignant transformation, and one case of endemetrioid borderline tumour.

Both the senior and junior sonologists were accurate in their subjective judgements of the following pathohistological types: benign lesions including mature teratoma in 60/83 cases (72.3%), haemorrhagic cyst in 13/15 cases (86.7%), simple cyst in 15/18 cases (83.3%), paraovarian cyst in 5/5 cases (100%), hydrosalpinx in 31/38 cases (81.6%), and endometriotic cyst of the ovary in 100/131 cases (76.3%); and malignant lesions including serous carcinoma in 19/21 cases (90.5%) and metastasis to ovary in 5/5 cases (100%).

### O-RADS classification

For the junior sonologist, the statistics of O-RADS classification 2 ~ 5 were analysed. Among the 332 patients (62.6%) with lesions classified as O-RADS 2, none had malignant pathological findings, resulting in a 0% malignancy risk rate. Out of the 82 patients (15.5%) with lesions classified as O-RADS 3, 3 had malignant pathological findings, resulting in a malignancy risk rate of 4%. Additionally, out of the 89 patients (16.8%) with lesions classified as O-RADS 4, 40 had malignant pathological findings, resulting in a malignancy risk rate of 45%. Last, out of the 27 patients (5.1%) with lesions classified as O-RADS 5, 17 had malignant pathological findings, resulting in a malignancy risk rate of 63%.

For the senior sonologist, the statistics of O-RADS classification 2 ~ 5 were analysed. Among the 335 patients (63.2%) with lesions classified as O-RADS 2, none had malignant pathological findings, resulting in a 0% malignancy risk rate. Out of the 82 patients (15.5%) with lesions classified as O-RADS 3, only 1 had malignant pathological findings, resulting in a malignancy risk rate of 1%. Additionally, out of the 79 patients (14.9%) with lesions classified as O-RADS 4, 34 had malignant pathological findings, resulting in a malignancy risk rate of 43%. Last, out of the 34 patients (6.4%) with lesions classified as O-RADS 5, 25 had malignant pathological findings, resulting in a malignancy risk rate of 74%.

Compared to the junior sonologist, the senior sonologist classified some lesions as being in a higher O-RADS category. One case of endometriotic cyst of the ovary and one case of fibroma were classified as O-RADS category 2 by the junior sonologist and as O-RADS category 4 by the senior sonologist. Additionally, one case of pelvic tuberculosis was classified as O-RADS category 2 by the junior sonologist and as O-RADS category 5 by the senior sonologist. Two cases of serous borderline tumour, one case of serous cystadenoma and one case of mature teratoma were classified as O-RADS category 3 by the junior sonologist and as O-RADS category 4 by the senior sonologist.

Compared to the junior sonologist, the senior sonologist classified some lesions as being in a lower O-RADS category. One case of endometriotic cyst of the ovary and one case of mature teratoma were classified as O-RADS category 4 by the junior sonologist and as O-RADS category 2 by the senior sonologist. One case of serous cystadenoma, one case of mature teratoma, two cases of endometriotic cyst of the ovary, one case of thecoma and one case of fibroma were classified as O-RADS category 4 by the junior sonologist and as O-RADS category 3 by the senior sonologist. One case of endometriotic cyst of the ovary was classified as O-RADS category 5 by the junior sonologist and as O-RADS category 2 by the senior sonologist. One case of gonadoblastoma was classified as O-RADS category 5 by the junior sonologist and as O-RADS category 3 by the senior sonologist.

Both the senior and junior sonologists diagnosed 36 patients as having O-RADS 4 lesions, indicating suspected malignancy. However, the histological results revealed benign pathology. These included one case of leydig cell tumours, 4 cases of mature teratoma, one case of serous surface papilloma, 4 cases of serous cystadenoma, one case of seromucinous cystadenoma, one case of pelvic tuberculosis, 2 cases of torsion of ovarian cyst, 2 cases of thecoma, one case of leiomyoma, one case of hydrosalpinx, 2 cases of fibroma, one case of serous adenofibroma, one case of adnexal inflammatory mass, one case of sclerosing stromal tumour, 12 cases of mucinous cystadenoma, and one case of endometriotic cyst of the ovary .

Both the senior and junior sonologists diagnosed 6 patients as having O-RADS 5 lesions, indicating malignancy. However, the histological results showed benign pathology. These included 1 case of microcystic stromal tumour, 1 case of thecoma, 1 case of fibroma, 1 case of mucinous cystadenoma, 1 case of an adnexal inflammatory mass and 1 case of pelvic tuberculosis. The O-RADS lexicon descriptions of adnexal lesions and the corresponding pathological results for both sonologists are presented in Table 2.

**Table 2:** Pathological results of O-RADS classification by sonographers with different seniority

O-RADS Score		Senior sonologist				Junior sonologist			
		All	Benign, n (%)	Malignant, n(%)	PPV	All	Benign, n (%)		Malignant, n(%)PPV
2	Non-simple unilocular cyst, smooth inner margin (< 10 cm)	5	5	0	0%	3	3	0	0%
	Classic Benign Lesions	211	211	0		218	218	0	
	Simple cyst (< 10 cm)	116	116	0		114	114	0	
3	Solid smooth, any size, CS = 1	3	2	1	4%	6	6	0	1%
	Multilocular cyst<10 cm, smooth inner wall, CS = 1–3	27	26	1		20	20	0	
	Unilocular cyst≥10 cm	35	35	0		32	32	0	
	Unilocular cyst, any size with irregular inner wall<3 mm height	0	0	0		6	6	0	
	Typical dermoid cysts, endometriomas, hemorrhagic cysts≥10 cm	17	16	1		18	17	1	
4	Solid smooth, any size, CS = 2–3	21	9	12	45%	16	8	8	43%
	Multilocular cyst, no solid component–Any size, smooth inner wall, CS = 4	0	0	0		1	1	0	
	Multilocular cyst, no solid component–Any size, irregular inner wall and/or irregular septation, any color score	6	3	3		7	7	0	
	Multilocular cyst, no solid component–≥10 cm, smooth inner wall, CS = 1–3	14	13	1		12	12	0	
	Multilocular cyst with solid component, any size, CS = 1–2	22	14	8		21	12	9	
	Unilocular cyst with solid component, any size, 0–3 papillary projections, CS = any	26	10	16		22	5	17	
5	Multilocular cyst with solid component, any size, CS = 3–4	3	2	1	63%	10	3	7	74%
	Solid smooth, any size, CS = 4	2	1	1		2	1	1	
	Solid irregular, any size, CS = any	2	1	1		5	1	4	
	Ascites and/or peritoneal nodules	19	5	14		16	3	13	
	Unilocular cyst, any size,≥4 papillary, CS = any	1	1	0		1	1	0	

### Comparison of diagnostic efficacy and evaluation of the consistency of the two diagnostic methods

The diagnostic efficacy of the O-RADS classification was found to be higher than that of the subjective judgement of the junior sonologist. The AUC for the O-RADS classification was 0.94 (95% CI 0.92–0.96), which was greater than that for the subjective judgement, which was 0.79 (95% CI 0.73–0.84), indicating statistical significance (*P* < 0.05). Furthermore, the O-RADS classification showed a significantly higher sensitivity, specificity, PPV, and NPV compared to subjective judgement.

The senior sonologist demonstrated higher diagnostic efficacy when using the O-RADS classification compared to their subjective judgement, with a statistically significant difference in the AUC (0.96 (95% CI 0.94 to 0.97) vs. 0.93 (95% CI 0.88 to 0.98), respectively) (*P* < 0.05). Compared to subjective judgement, the O-RADS classification showed a higher sensitivity and NPV, but the specificity and PPV were lower.

**Fig. 2:**
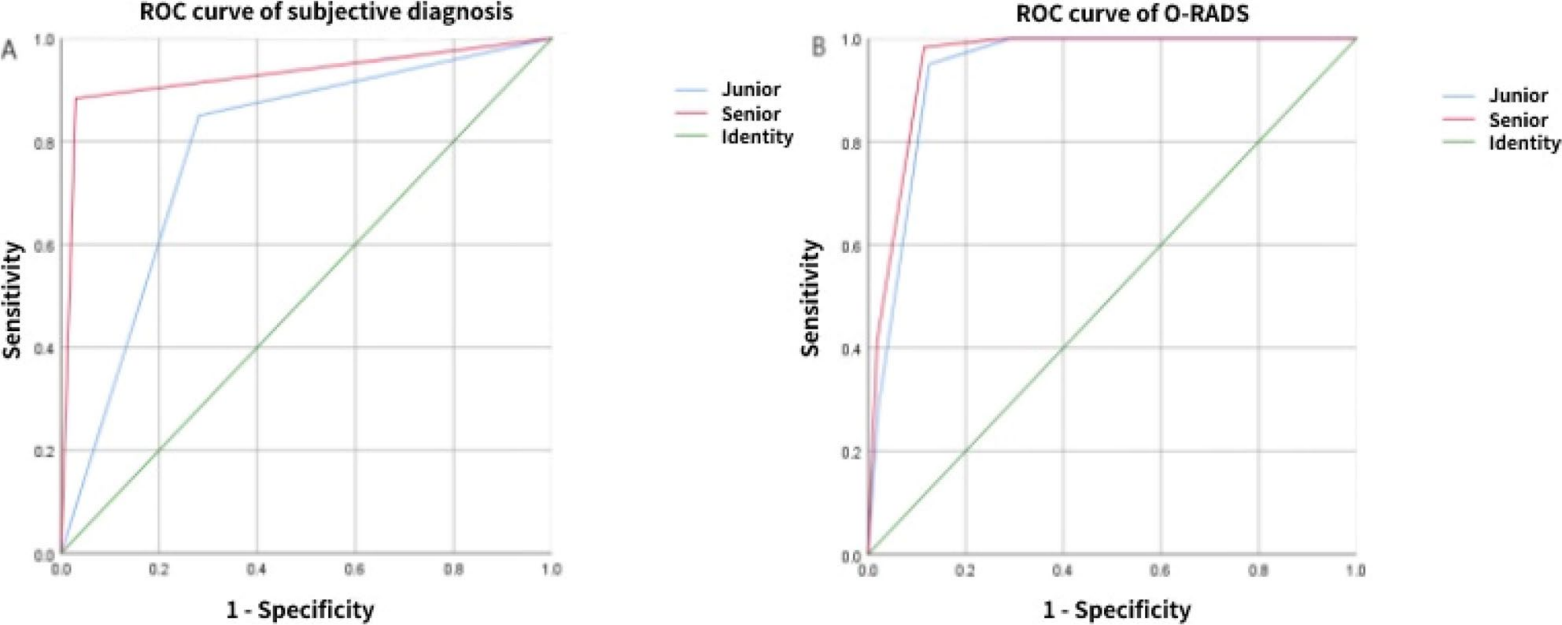
Subjective diagnosis (**A**) and O-RADS classification (**B**) AUC by senior and junior sonologists

Comparison of diagnostic efficacy and evaluation of consistency among sonologists with varying levels of experience For the senior sonologist, the diagnostic efficacy of subjective judgement and O-RADS classification was higher than that of the junior sonologist, and there was a statistically significant difference (*P* < 0.05). For subjective judgment, the diagnostic agreement between the junior and senior sonologists showed a lower Kappa value of 0.34 (*P* < 0.05). The diagnostic agreement between the junior and senior sonologists for O-RADS classification was improved compared to that for subjective judgement, with a Kappa value of 0.91 (*P* < 0.05). This is illustrated in Fig. 2; Tables 3, 4 and 5.

**Table 3:** Comparison of the diagnostic performance of two diagnostic methods of different sonologists

	Sensitivity (%)	Specificity (%)	PPV	NPV	AUC(95%CI)	*P*
Subjective diagnosis of junior sonologist	85(76 ~ 93)	72(69 ~ 77)	28(25 ~ 39)	97(95 ~ 99)	0.79(0.73 ~ 0.84)	<0.05
Subjective diagnosis of senior sonologist	88(77 ~ 95)	97(95 ~ 98)	79(67 ~ 88)	98(97 ~ 99)	0.93(0.88 ~ 0.98)	
O-RADS of junior sonologist	95(86 ~ 99)	87(84 ~ 91)	49(41 ~ 60)	99(98 ~ 100)	0.94(0.92 ~ 0.96)	<0.05
O-RADS of senior sonologist	98(90 ~ 100)	89(85 ~ 91)	52(43 ~ 62)	100(98 ~ 100)	0.96(0.94 ~ 0.97)	

*AUC* Area under the curve, *CI* Confidence interval, *PPV* Positive predictive value, *NPV* Negative predictive value

**Table 4:** Consistency analysis of subjective diagnosis of different sonologists

Senior sonologist	Junior sonologist		
	Malignant	Benign	All
Malignant	58	125	183
Benign	9	338	347
All	67	463	530

Kappa = 0.34, *P*<0.05

**Table 5:** Consistency analysis of subjective diagnosis of different sonologists

Senior sonologist	Junior sonologist				All
	O-RADS US 2	O-RADS US 3	O-RADS US 4	O-RADS US 5	
O-RADS US 2	327	2	2	1	332
O-RADS US 3	5	73	4	0	82
O-RADS US 4	2	6	71	10	89
O-RADS US 5	1	1	2	23	27
All	335	82	79	34	530

Kappa = 0.91,*P*<0.05

## Discussion

**Fig. 3:**
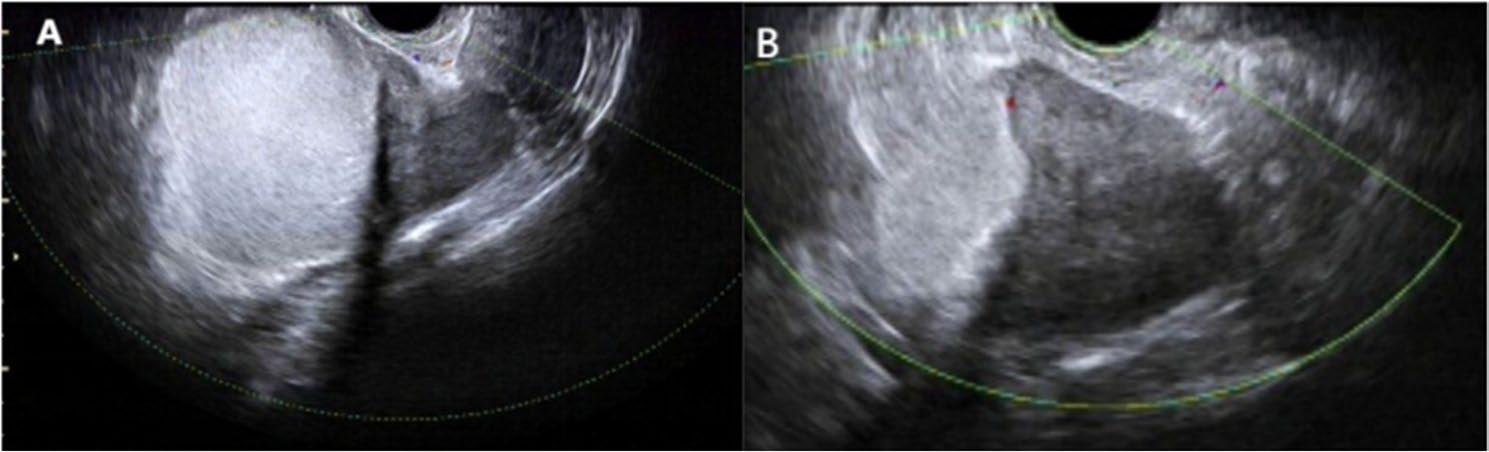
A 58-year-old woman had been in menopause for 2 years. She had experienced intermittent right lower abdominal pain for 1 month. The postoperative pathological examination confirmed teratoma with malignant transformation. **A** Most of the lesions exhibited typical teratoma characteristics with hyperechoic components, while a small portion displayed irregular hypoechoic areas. **B** Punctate blood flow was observed in this particular region of the lesion

The junior sonologist showed similar subjective judgement to the senior sonologist in evaluating missed cases. Additionally, the distribution of pathohistological types of missed cases was consistent across different groups. One case of teratoma with malignant transformation was missed in this study. The patient was a 58-year-old woman who had been in menopause for 2 years. She came to the hospital due to intermittent right lower abdominal pain that had persisted for 1 month. Ultrasound examination revealed a lesion measuring approximately 11.1 cm x 9.2 cm x 11.9 cm. Most of the lesions exhibited typical teratoma characteristics with hyperechoic components, while a small portion displayed irregular hypoechoic areas. Additionally, punctate blood flow was observed in this particular region of the lesion. However, the sonologist overlooked the presence of point-like blood flow, mistook the low echo portion for a cystic structure, and consequently missed the diagnosis. The preoperative CT and MRI scans showed the presence of a teratoma, with no signs of enhancement or malignant alterations. However, the postoperative pathological examination confirmed teratoma with malignant transformation. The image information is shown in Fig. 3.

According to the literature, the malignant transformation of ovarian mature teratomas is very uncommon, with an incidence rate of approximately 0.17% to 2%, representing just 1.5% to 2.3% of all primary ovarian malignant tumours [18, 19]. The predominant histological subtype of this condition is squamous cell carcinoma, which constitutes approximately 80% of cases [20]. Some researchers have proposed considering teratoma malignancy in patients over 40 years old, particularly if the tumour measures > 10 cm and/or includes a solid component [21]. In addition, sonologists should carefully determine whether septations and papillae are combined in cystic lesions and adequately assess the blood flow signal to avoid missing the diagnosis of junctional or malignant ovarian epithelial tumours. This can be accomplished by narrowing the sampling frame to include only the papillary echoes and observing blood flow with power Doppler or microvascular flow.The research by Di Legge et al. also confirms that even for tumors of small size, ultrasound examination can achieve accurate diagnosis [22, 23]. 

**Fig. 4:**
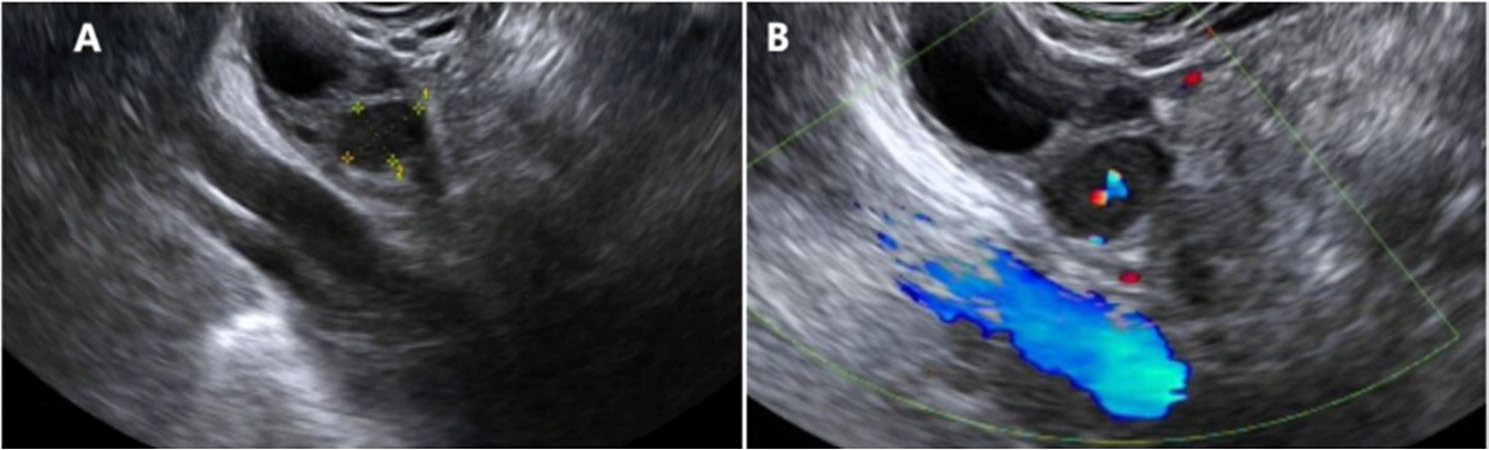
A 27-year-old patient, with right lower abdominal pain for 2 weeks. **A** Ultrasound revealed a rounded weakechogenicity in the right ovary, with clear and regular borders. **B** regular striped blood flow signals were observedboth in the periphery and inside of the mass the postoperative pathological examination confirmed cellular fibroma

Another missed case of cellular fibroma, a less common histological type, involved a 27-year-old patient who came to the clinic due to a pelvic mass that had been present for 5 months and the presence of right lower abdominal pain for 2 weeks. Ultrasound revealed a rounded weak echogenicity in the right ovary, measuring approximately 1.3 cm x 1.0 cm x 1.2 cm, with clear and regular borders. Additionally, regular striped blood flow signals were observed both in the periphery and inside of the mass, and the image information is shown in Fig. 4. Imaging did not show typical malignant features, resulting in a missed diagnosis; the patient’s preoperative carcinoembryonic antigen tests were normal, and no other imaging tests were performed.

In terms of subjective judgement and misdiagnosed cases, the subjective judgement of the senior sonologist was significantly better than the subjective judgement of the junior sonologist(senior specificity 97% vs. junior 72%). When the histological type of a teratoma is a monolayer (such as struma ovarii), ultrasound shows a solid lesion with abundant blood flow, which is easily misdiagnosed as malignant. In this group, there was one misdiagnosed case of ovarian struma ovarii. In addition, teratomas in adolescent females frequently manifest as larger and more intricate lesions, leading to potential misdiagnosis as malignant tumours. Among the misdiagnosed cases of teratoma within this group, 4 out of the 23 cases were identified in adolescents.

The combination of residual normal ovarian tissue with teratoma lesions makes it difficult to distinguish from a solid lesion, often leading to misdiagnosis. When the history of endometriotic cyst of the ovary is long, large wall echoes, thick cyst contents, and linear septa will be present, which can be easily misdiagnosed as solid lesions. Atypical hydrosalpinx, which can involve the accumulation of blood or pus and the development of complex echoes, is prone to misdiagnosis. When tubulitis involves the ovary and ovarian cysts, incomplete segregation of the imaging characteristics is easy to misdiagnose. Regarding torsion of ovarian cyst, normal ovarian tissue is difficult to identify, the echogenicity of the lesion is disturbed, and the blood flow signal is atypical, which makes it easy for a physician to misdiagnose the lesion as malignant when the patient’s medical history is ignored. Larger serous cystadenoma and mucinous cystadenoma are easily misdiagnosed as malignant when combined with segregation and poor translucency.

The abovementioned pathological tissues have complex histological compositions, and the sonographic images of these tissues are often atypical. Moreover, the medical history of these patients is long, and the ultrasound manifestations are diverse and complex, showing characteristics of the same image with different diseases and the same disease with different images. This makes subjective diagnosis by sonologists difficult, especially junior sonologists. Correctly distinguishing the images of different diseases is the key to improving diagnostic efficiency. Experienced ultrasound practitioners often encounter instances of subjective misdiagnosis, primarily characterized by imaging characteristics that resemble malignant tumours. These characteristics include larger volumes, the presence of accumulated fluid in the pelvis and abdomen, multicystic lesions with solid components, complex echoes, and abundant blood flow signals.

Compared with the senior sonologist, the junior sonologist who analysed the data in this study had 3 years of experience, less exposure to different types of diseases and had treated fewer patients. For some typical lesions, the junior sonologist had a good diagnostic accuracy, including for most benign lesions with typical ultrasound manifestations, such as the short-line sign or dough sign of teratomas, and the internal echo of endometriotic cyst of the ovary with a ground-glass appearance. Typical malignant lesions have larger papillary echoes and abundant blood flow signals and are often accompanied by pelvic or abdominal effusion. Following training, junior sonologist is capable of accurately and reliably identifying the infiltration of ovarian cancer across various anatomical sites [24]. However, the diagnostic accuracy for atypical lesions and some complex lesions is low. For the preoperative detection of borderline ovarian tumor could have a considerable positive effect on the management of the disease in younger women, all patients of childbearing age in this study underwent ovarian cystectomy. Moro et al. confirm that there was an overlaping ultrasound appearance between borderline ovarian tumor and non-invasive low grade serous ovarian carcinoma, both presenting as cysts with papillary projections [25–27]. Junior sonologists should pay attention to some borderline lesions and, if necessary, consult with senior sonologists in order to prevent diagnostic errors that may lead to excessive treatment.

In this study, the malignant risk judged by sonologists with different years of experience using O-RADS categories 2–5 was consistent with the literature. For the two sonologists, the malignant risk rates among O-RADS categories 2–4 were basically the same, and only the malignant risk rate of O-RADS category 5 determined by the senior sonologist was slightly higher than that determined by the junior sonologist. This indicates that the senior sonologist classified more malignant lesions as O-RADS category 5. However, the overall consistency of O-RADS classification between the two sonologists was quite good, with a kappa value of 0.91. Similar to the findings of Yang, Lai et al. [28, 29], the misclassification of the lesion by the ultrasound physician led to an erroneous determination of its malignant risk.

Some typical benign lesions, such as endometriotic cyst of the ovary and mature teratomas of the ovaries, are misclassified by junior sonologists, leading to an overestimation of malignant risk. The reason is that when typical benign features are encountered, standardized ultrasound terminology should be used to make corresponding characteristic diagnoses. The colour score is not used to evaluate such lesions. For example, endometriotic cyst of the ovary should be described using terms such as ground-glass or uniformly hypoechoic; mature teratomas of the ovaries should be described using terms such as echogenic components, echogenic lines or dots, and floating echogenic spherical structures. If ultrasound descriptive terminology is not standardized, it may lead to misdiagnosis and inappropriate management. Therefore, the O-RADS Consensus Guidelines for Ultrasound Risk Stratification and Management emphasize the importance of standardized ultrasound terminology for the description of some typical benign ovarian-adnexal lesions.

When there are septations within some teratoma lesions, the O-RADS category tends to be higher. In clinical diagnosis, high echogenicity components and typical teratomacharacteristics characteristics without blood flow can be used as the basis for judgement to improve the accuracy of O-RADS classification. When a physician considers a teratoma based on imaging features, but there is abundant blood flow, it is necessary to consider whether ovarian thyroid nodules are also present. At the same time, atypical mature teratomas and endometriotic cyst of the ovary cannot be classified as typical benign lesions but should be classified according to ultrasound terminology.

The reason for the inconsistency in O-RADS classification between senior sonologist and junior sonologist is the inconsistency in determining whether hypoechoic and hyperechoic components are solid, whether the margins of solid lesions are smooth, the cyst wall and intracystic septations of multiple single-cyst cysts, and the blood flow score. These factors lead to errors in O-RADS classification judgements and are real challenges for sonologists in daily practice.

Some lesions are more likely to be classified in a higher O-RADS malignancy category by both junior and senior sonologists. For instance, Fibromas accompanied by Meigs syndrome often present with ascites, the O-RADS category is higher. When ultrasound physicians subjectively diagnose fibroma, their subjective assessment should indicate a tendency towards a benign condition in the ultrasound report. A multilocular endometriotic cyst of the ovary with acute haemorrhage can also result in a high O-RADS classification. Sonologists should consider patients’ medical histories and laboratory test indicators to make a thorough assessment. Sonologists can use contrast-enhanced ultrasound to determine whether an AM is cystic or solid in nature. This is important because during an abdominal ultrasound examination, it is easy to underestimate the blood flow of the AM. Misinterpreting nonblood flow high echogenic components as solid, such as a high echogenic teratoma, may lead to misjudging it as malignant.

For inflammatory lesions, the ultrasound characteristics often show complex echoes, irregular solid edges, and abundant blood flow signals, which make them easy to assess as malignant; when classifying these lesions, they should be classified according to the patient’s medical history, changes in the mass over time, and clinical symptoms. Sonologists may overestimate the risk of malignancy if they mistakenly interpret a small amount of ovarian tissue at the edge of a complex cyst as a solid portion of the cyst. Furthermore, the overestimation of malignancy risk can occur due to the misinterpretation of incomplete septations as complete septations or the misinterpretation of multiple adjacent cysts as multilocular cysts. In clinical practice, sonologists should fully utilize the advantages of multiangle scanning with intracavitary probes to obtain ideal ultrasound images using the cross method for more accurate assessments.

In this study, some lesions were categorized as O-RADS 4 because of multilocular cysts, with or without solid components, and a blood flow score of 1–3. However, the postoperative pathological results confirmed that these lesions were benign, leading to an increase in the misdiagnosis rate. Therefore, sub-classifying lesions in the O-RADS category 4 can improve diagnostic efficiency [30].

In the current guidelines, the classification of complex ovarian cysts with solid components into O-RADS categories 4 or 5 relies on the cut-off value of the blood flow score. Consequently, the blood flow score plays a crucial role in O-RADS classification. However, accurately evaluating the blood flow signals of ovarian lesions is often challenging due to their deep location within the pelvic cavity. Furthermore, the availability of appropriate ultrasound equipment poses an additional obstacle. Typically, tumour specialty research institutions possess gynaecological-specific high-end colour Doppler ultrasound equipment, while most institutions lack such resources and instead utilize low-end or whole-body machines. As a result, the sensitivity of blood flow evaluation is generally poor, leading to potential misjudgement of the blood flow score and subsequently lower malignant risk assessment for certain complex ovarian cysts with solid components.

The sensitivity of the senior sonologist’s subjective judgment (88%) was slightly higher than that of the junior sonologist (85%), and there was still room for improvement. However, the specificity of the senior sonologist (97%) was significantly higher than that of the junior sonologist (72%). This suggests that the clinical experience of senior sonologists is advantageous in accurately excluding the malignant risk of benign lesions and achieving a higher PPV. On the other hand, the PPV of the junior sonologist’s subjective judgement was notably low, at only 28%. This can lead to excessive diagnosis and treatment in clinical practice. Consequently, it is imperative to implement measures aimed at enhancing the diagnostic positive predictive ability of sonologists with different experience levels.

The evaluation of prediction models for malignant tumours often relies on diagnostic sensitivity as a crucial indicator. Our study found that the AUC of the O-RADS classification, when compared to subjective assessment, was significantly higher for ultrasonologists with different levels of experience (*p* < 0.05), consistent with the findings of Guo [31]. Specifically, the diagnostic sensitivity and specificity of the junior sonologist were notably improved, with the PPV increasing from 28% to 49%. This suggests that the O-RADS classification system is particularly beneficial for junior sonologists. The detailed definitions of imaging features and ultrasound descriptive terms for benign and malignant lesions provided by the O-RADS enhance the detection rate of malignant masses and mitigate the potential consequences of missed diagnoses. However, importantly, in our study, the PPV of the O-RADS diagnosis was relatively low for sonologists with varying years of experience. This can be attributed to the fact that the optimal cut-off value of the O-RADS ROC curve for predicting malignancy in our study was an O-RADS category > 3.5. Simultaneously, the uneven risk of malignancy among lesions in O-RADS category 4 led to the misclassification of benign lesions as malignant in all four categories.

Consistent with the findings of Pelayo’s study [32], The ability to differentiate between benign and malignant lesions is significantly improved by the O-RADS classification, as evidenced by the 7 cases of subjective missed diagnoses by the senior sonologist, all of which were classified as O-RADS 4 or 5. However, the O-RADS classification was also higher in the 14 cases of subjective misdiagnosis, indicating a certain difficulty in diagnosing these lesions subjectively and according to the O-RADS classification. This difficulty primarily arises in benign sex cord-stromal tumours, including thecoma and atypical teratomas, due to the presence of accumulated pelvic or abdominal fluid or rich blood flow signals. These factors lead to a subjective diagnosis of malignancy and an O-RADS classification of O-RADS 5. Therefore, further summarization of clinical experience is necessary for lesions of this histological type, and the use of other imaging methods, such as contrast-enhanced ultrasound, may be considered where available.

In this study, the subjective judgements of the senior and junior sonologists exhibited poor consistency, as indicated by a kappa value of 0.34. This lack of agreement can be attributed to the fact that the junior sonologist demonstrated higher diagnostic accuracy for typical benign and malignant lesions. However, they tended to overestimate the malignancy of atypical lesions and difficult cases, resulting in the misclassification of some benign lesions as malignant. Conversely, when utilizing the O-RADS classification system, there was good consistency between the senior and junior sonologists, as evidenced by a kappa value of 0.91. The implementation of standardized ultrasound terminology enables sonologists to provide more detailed and precise descriptions of lesions, thereby facilitating more objective assessments of lesion benignity or malignancy. Furthermore, importantly, in this retrospective study, both sonologists evaluated the lesions based on images acquired by senior sonologists. Additionally, the research team conducted prescreening of imaging data and other pertinent information, which may have contributed to an overestimation of the consistency between observers and the diagnostic performance of the junior sonologist utilizing the O-RADS classification system.

There are several limitations associated with this study. First, the use of retrospective studies restricted the evaluation of lesions to existing ultrasound images, which may have resulted in the misinterpretation of features such as boundaries or blood flow. Second, the malignancy rate observed in the sample of this study was relatively low (11.3%) compared to previous studies [33], where the malignancy rate ranged from 27.5% to 28.8%. Third, the O-RADS management recommendations were not validated in this study. Last, importantly, this study was conducted at a single centre, and therefore, further validation is needed through large-scale multicentre studies.

## Conclusions

Overall, it plays an important role in providing a reference for ultrasound doctors in diagnosing AMs and promoting the application of the O-RADS in clinical practice. This will assist clinicians in accurately diagnosing and treating patients with ovarian tumours.

### O-RADS outlook

1. This study found that the requirement of ultrasound image quality is extremely high of the O-RADS, especially the characteristics of septa, blood flow, and solid components, which are easily limited or misleading by ultrasound images. Therefore, to improve the accuracy of O-RADS classification, standardized collection of ultrasound image data is necessary.Such as mandate power Doppler for all complex cysts; archive cine loops ≥ 10 s.
2. Even with O-RADS-related training, there are still large differences in senior and junior sonologists’ understanding of the guidelines. Therefore, the O-RADS may need to be standardized, and training guidelines or courses should be provided to promote wide clinical application.
3. More prospective, multicenter studies, particularly those involving large datasets across different regions and ethnic groups, should be conducted to validate the universal applicability and clinical value of the O-RADS system.
4. O-RADS 4 lesions are commonly misclassified, and there is significant overlap between the four categories of benign and malignant lesions. In clinical practice, sonologists should indicate their subjective tendency towards benign or malignant lesions within these categories in the report. This information helps clinicians in their subsequent management decisions.
5. The blood flow score is an important index in O-RADS classification, but it is difficult for sonologists to obtain true and accurate blood flow signals of pelvic masses due to the deep pelvic position, thickness of abdominal fat and insufficient resolution of the instrument. In clinical application, it is recommended that all gynaecological pelvic AMs be evaluated by power Doppler flow imaging, which should be standardized in the form of guidelines.

### Statistical analysis

The data were statistically analysed using SPSS 26.0. Continuous variables are presented as the mean ± standard deviation, while categorical variables are expressed as counts or percentages. Categorical variables were compared using a chi-square test. Interobserver agreement was assessed using the kappa value (к). The following kappa ranges were used: к ≤ 0.20 (poor agreement), к of 0.21–0.40 (fair consistency), к of 0.41–0.60 (moderate consistency), к of 0.61–0.80 (good agreement), and к of 0.81-1.00 (almost perfect agreement). Using pathological results as the gold standard, the receiver operating characteristic (ROC) curve was plotted. The area under the curve (AUC) and Youden index were calculated. The largest Youden index was selected as the optimal cut-off value, and the sensitivity, specificity, positive predictive value (PPV), and negative predictive value (NPV) were determined. Statistical significance was determined at the *P* < 0.05 threshold.

## Data Availability

The datasets used and/or analysed during the current study are available from the corresponding author on reasonable request.

## Abbreviations

AMs: Adnexal masses
IOTA: International Ovarian Tumor Analysis
SR: Simple Rules
GI-RADS: Gynaecologic Imaging Reporting Data System
SR-Risk: Simple Rules-Risk Model
ACR: American College of Radiology
O-RADS: Ovarian Adnexal Imaging Reporting and Data System
WHO ICOT: World Health Organization International Classification of Ovarian Tumors
ROC: Receiver operating characteristic
AUC: Area under the curve
PPV: Positive predictive value
NPV: Negative predictive value
